# Enhancing Preschoolers’ Motor Creativity Through Playfulness and Social Engagement

**DOI:** 10.3390/children12080969

**Published:** 2025-07-23

**Authors:** Despoina Ourda, Eleni Polyzoudi, Athanasios Gregoriadis, Vassilis Barkoukis

**Affiliations:** 1 Department of Physical Education and Sport Science, Aristotle University of Thessaloniki, 54124 Thessaloniki, Greece; despoino@phed.auth.gr (D.O.); elenipolizoudi@gmail.com (E.P.); 2 Department of Early Childhood Education and Care, Aristotle University of Thessaloniki, 54124 Thessaloniki, Greece; asis@nured.auth.gr

**Keywords:** innovation, movement, spontaneity, social profile, social status, kindergarten

## Abstract

**Background/Objectives**: Motor creativity is a vital component of preschoolers’ growth and development. However, its underlying determinants remain largely underexplored. This study investigates the interrelationship among playful behavior, social profile, and motor creativity in preschool children, emphasizing its implications for holistic health and development. **Methods**: A total of 200 children and their kindergarten teachers from Greece participated in the study. The Children’s Playfulness Scale (CPS) was employed to assess the children’s playfulness, while a sociometric test was used to evaluate their social standing within peer groups. Motor creativity was measured through TCAM, a performance-based test focusing on fluency, imagination, and originality. **Results**: The findings revealed that the dimensions of playful behavior, particularly motor and social playfulness, significantly and positively influenced motor creativity, a core component of physical and mental health in early childhood. Conversely, certain aspects of social behavior had a negative association with imaginative capacities. **Conclusions**: The study underscores the critical role of movement-based playful activities in fostering children’s physical, emotional, cognitive, and social health. It highlights the need for educators to design developmentally appropriate motor play activities that cultivate creativity and social integration, promoting a balanced and health-oriented early education framework. The results contribute to educational policy and practice by reinforcing the importance of structured motor play in supporting preschoolers’ well-being and comprehensive development.

## 1. Introduction

Play has held a central role in children’s education, serving as both a primary activity and a vital medium for learning, especially among preschoolers. Through play, children experience joy, heightened interest, and active engagement, leading to meaningful learning experiences. Beyond its educational value, play is essential for children’s overall health and development. Ginsburg et al. [1] emphasized that play contributes significantly to the cognitive, physical, social, and emotional well-being of children and youth. Engaging in play also promotes physical health by enhancing motor skills, coordination, and overall fitness. Furthermore, play supports mental health by reducing stress, anxiety, and depression. In addition, it aids in developing emotional and social skills and helps children cope with stress and fosters resilience. Thus, children’s playful behavior can set the basis for their physical and social health and welfare. Importantly, peer-playing has been suggested as an important means for the social development and welfare of the child [2]. In preschool education, motor creativity can be an index of effective integration of play into the child’s socialization and life [3]. Still, the association of playfulness and social engagement in kindergartners with motor creativity has not been thoroughly investigated.

### 1.1. Motor Creativity as a Foundation for Early Childhood Development

Motor creativity refers to the capacity to generate novel, original, and functional motor patterns, often in response to open-ended tasks. It is increasingly recognized as a central component of physical literacy and a key indicator of adaptive movement behavior in early childhood [4,5]. The preschool years constitute a sensitive period for the development of motor creativity, as children explore movement possibilities through play, imitation, and experimentation. This process is associated not only with biomechanical efficiency but also with cognitive and affective indicators, which are all critical to overall child development [6,7,8]. Recent research indicates that the development of motor creativity contributes positively to various health outcomes in preschoolers. For instance, children who demonstrate higher levels of motor creativity are more likely to engage in health-promoting behaviors, such as physical activity and positive attitudes towards healthy nutrition, which are associated with maintaining healthy weight trajectories [9]. Movement tasks that encourage creative exploration, such as improvised games, outdoor play, and object control, support motor coordination and cognitive development, like verbal intelligence, attention span, memory retention, and problem-solving skills [10,11]. As Garaigordobil et al. [11] indicated, movement tasks foster assertive cognitive strategies and prosocial problem-solving, strengthen relationships and positive group communication, and enhance verbal intelligence, creative thinking (both verbal and visual), and traits associated with a creative personality. They are also associated with overall cognitive abilities [12], suggesting that enriched physical education lessons, including cognitively challenging tasks, play a role in mental and physical health as well. More specifically, Pesce et al. [12] found that children in the enriched intervention group showed greater improvements in motor coordination and cognitive processing.

In addition to physical health, research indicates that fostering motor creativity in children contributes significantly to their social–emotional growth and enhances essential skills such as empathy, emotional stability, communication and cooperation skills, self-concept, and peer image [10,11], which can contribute to long-term adherence to physically active lifestyles. This evidence indicates that movement tasks contribute to the enhancement of positive social behaviors, the reduction of negative ones, and the improvement of self-concept, peer relationships, and emotional stability [11]. Furthermore, creative movement provides an outlet for emotional expression and identity development, allowing children to explore their abilities in a low-pressure, playful environment [11]. These psychosocial dimensions are particularly important during the preschool years, when self-concept and emotional coping strategies are forming. Recent research supports the idea that motor creativity, as expressed through open-ended, imaginative movement tasks, is deeply intertwined with social engagement, as children learn to adapt, respond, and co-create in group contexts [10].

### 1.2. Playfulness in Early Childhood

Playfulness is increasingly recognized as a critical personality trait and behavioral disposition that supports holistic development [13]. Defined by dimensions such as motor spontaneity, cognitive inventiveness, social engagement, joy, and humor [14], playfulness enables children to explore their environment, test boundaries, and express themselves in imaginative and divergent ways. As research has shown, playfulness is not only associated with positive emotional states [15], but it also predicts higher levels of physical activity and engagement in motor tasks [16]. Research findings underscore the role of playfulness as a dynamic mechanism that facilitates both motor development and psychosocial well-being in young children [16,17].

Playful children, due to their enhanced spontaneity, imaginative flexibility, and willingness to explore, tend to demonstrate superior performance in motor creativity tasks [18]. As children play, they are more inclined to take initiative, break routine, and test alternatives; they are also more likely to excel in activities requiring creative problem-solving through movement [19]. Existing studies have confirmed that structured, yet flexible, movement programs based on play principles improve preschoolers’ creative fluency and motor originality [18]. In addition to that, engaging in playful, movement-rich environments contributes significantly to children’s health, particularly by promoting physical fitness, mental resilience, and emotional regulation [17,20]. Indicatively, Adamopoulou et al. [17] indicated that an exercise program with modified fairytales improved children’s motor proficiency. Studies suggest that higher levels of playfulness correlate with increased participation in moderate-to-vigorous physical activity, leading to improved motor competence and reduced risk of sedentary behavior [16,21]. For instance, Fernandez et al. [21] noted that following an intervention, children’s participation in physical activities during recess was increased. Beyond physical health, playful children are also better equipped to manage stress, demonstrate agency and empathy, and engage in cooperative peer interactions [22,23], all critical indicators of social and emotional health. These effects are particularly pronounced in inclusive and diverse early childhood settings, where playful engagement fosters peer acceptance and positive classroom climates [22].

### 1.3. The Present Study

Motor creativity in preschoolers is integral to their overall well-being and development. Engaging in activities that promote motor creativity, such as imaginative play, dance, and exploratory movement, enhances not only physical skills but also cognitive and emotional growth. Research indicates that such creative motor activities support the development of problem-solving abilities, self-expression, and adaptability, which are crucial for children’s well-being. Yildirim and Yilmaz [24] found that promoting creativity in early childhood education was positively associated with children’s ability to express emotions and thoughts effectively, thereby contributing to their social and emotional development. Furthermore, the relationship between motor skills and social–emotional functioning has been explored in various studies. While some research suggests a weak correlation between social–emotional functioning and motor skills in typically developing preschool children [25], other studies emphasize the importance of creative play in enhancing social skills and emotional regulation [10,11]. For example, engaging in free play with peers has been associated with higher levels of social skills and self-regulation, as well as lower levels of aggression and adjustment problems. These findings underscore the multifaceted benefits of motor creativity in supporting preschoolers’ well-being and welfare [26]. Research has consistently shown that playful children are more likely to participate in creative movement tasks and demonstrate higher motor fluency and originality [18]. In this sense, playfulness can be viewed as a behavioral catalyst for divergent thinking in motor domains, as it promotes flexibility, risk-taking, and initiative, all essential components of creative motor performance.

Social context also plays a pivotal role in children’s creative development. Children’s sociometric status reflects the degree to which a child is integrated into or excluded from peer interactions. Children with high social engagement tend to participate more in group play, role-taking, and cooperative activities, contexts that are fertile ground for the co-construction of creative motor actions [10,11,22,23]. The interaction between playfulness and social profile can reflect a synergistic nature of development in early childhood. Motor creativity does not emerge in isolation but through complex transactions between the child’s internal dispositions (e.g., playfulness) and external social experiences (e.g., peer interactions). According to Vygotskian principles and dynamic systems theory, creativity in physical actions is socially mediated and scaffolded by opportunities for exploration within secure and socially responsive environments.

Despite the well-documented benefits of play and movement for children’s development [27], limited research has systematically examined how specific dimensions of playfulness and social integration relate to motor creativity in early childhood. While previous studies have highlighted the importance of creative motor tasks for physical, cognitive, and emotional development [4,10,11], most have focused on general motor competence or unidimensional aspects of play. Additionally, the role of the child’s social standing within peer groups, a potentially influential contextual factor, has received little attention in relation to motor creativity. To date, few empirical studies have attempted to simultaneously examine how behavioral dispositions like playfulness and social positioning interact to influence creative movement outcomes in preschool-aged children. This study addresses this gap by investigating how different dimensions of playful behavior and peer-related social status predict motor creativity, thereby offering a more comprehensive understanding of the factors that support holistic development during the critical preschool years. Grounded in developmental and educational psychology, the present study posits that playfulness, as a behavioral disposition, and social profile, as a contextual–social variable, are critical predictors of motor creativity.

## 2. Materials and Methods

### 2.1. Participants

A total of 20 kindergarten classes were selected to take part in the study. After an open invitation disseminated via social media and networks of kindergarten teachers, 20 educators answered within the specified timeframe and expressed interest in taking part in the study. The final sample comprised 200 children (100 boys and 100 girls), aged 4 to 5 years, with a mean age of 63.6 months (SD = 6.5). The children were enrolled in both public and private kindergartens in Thessaloniki, Greece.

### 2.2. Measures

*Playfulness*: To assess playful behavior, the Greek adaptation of the Children’s Playfulness Scale (CPS) was used [18]. The CPS is a 5-point Likert-type questionnaire comprising 23 items, rated on a scale from 1 (does not sound at all like the child) to 5 (sounds exactly like the child), based on the frequency of observed behaviors [28]). The questions focus on the children’s behaviors during play, and responses were completed by kindergarten teachers following direct observation of each child. The CPS is designed to evaluate five core dimensions of playfulness: physical spontaneity (e.g., “The child is physically active during play”), social spontaneity (e.g., “The child is willing to share toys”), cognitive spontaneity (e.g., “The child invents his/her own toys”), manifest joy (e.g., “The child expresses enjoyment while playing”), and sense of humor (e.g., “The child teases others playfully”). The Cronbach alphas were adequate for all subscales (alphas ranged from 0.68 to 0.80; see Table 1).

*Motor creativity:* To assess motor creativity, the Greek adaptation of Torrance’s Thinking Creatively in Action and Movement (TCAM) was used [29]. The TCAM includes four structured motor activities designed to evaluate three core components of motor creativity: fluency, originality, and imagination. In Activity 1, children are asked to travel a distance using as many different movement patterns as possible. Verbal, motor, or combined responses are accepted. The researcher encourages the child to express themselves freely and records all responses. This activity is used to assess both fluency and originality. In Activity 2, children are asked to physically represent various scenarios using their bodies, including a tree in strong wind, a hopping rabbit, a swimming fish, a crawling snake, a car driver, and themselves pushing an elephant. The researcher evaluates each representation using a 5-point Likert scale (1 = no movement/imitation, 3 = satisfactory performance, 5 = excellent representation), focusing on the dimension of imagination. Activity 3 involves placing paper cups into a basket in as many different ways as possible. Both motor and verbal responses are recorded and scored for fluency and originality. In Activity 4, children are asked to imagine alternative uses for the glass used in the previous activity and to verbalize their ideas. Their responses are assessed for fluency and originality. The scoring for fluency includes the total number of motor and verbal responses in Activities 1, 3, and 4 (score range: 0–40 per activity). For originality, each motor or verbal response in Activities 1, 3, and 4 is scored for uniqueness (0–3 points for Activities 1 and 4; 0–4 points for Activity 3) using Torrance’s originality rating chart. Lastly, the score for imagination is the sum of the scores from Activity 2, with each scenario rated from 1 to 5. The final motor creativity score is calculated by converting the raw scores for fluency, originality, and imagination using age-adjusted norms provided in Torrance’s scoring table. This ensures that children’s performance is interpreted in relation to developmental expectations.

*Social Profile*: To assess the social profile of each child, Moreno’s [30] sociometric test was employed. The test was administered individually in the form of a structured interview. Each child was asked to imagine going on a school bus field trip and to indicate up to three classmates they would prefer to sit near, and up to three they would prefer to sit away from. To ensure response accuracy, each verbal response was verified by having the child point to a corresponding photograph of the peer mentioned. A sociometric matrix was then created for each class, consisting of a double-entry table with children’s names listed in both rows and columns. This matrix recorded the number of positive (preferred peers) and negative (non-preferred peers) nominations each child received. Based on the frequency and pattern of these nominations, the children were classified into five sociometric status categories, including popular students [i.e., those receiving several positive choices and few to no negative ones (e.g., 6–0, 7–0, 5–1)], average students [i.e., those receiving moderate positive choices with few or occasional negative ones (e.g., 3–1, 3–0, 4–2)], controversial students [i.e., those receiving several positive and negative choices (e.g., 3–3, 4–3, 5–4)], rejected students [i.e., those receiving predominantly negative choices (e.g., 0–6, 0–7, 1–7, 2–9)], and neglected students [i.e., those receiving few or no positive or negative choices (e.g., 0–0, 1–0, 1–1)]

### 2.3. Procedure

The study design was cross-sectional, and ethics approval was granted from the “Committee of Ethics in Research of the Department of Physical Education and Sport Science” of the Aristotle University of Thessaloniki. Prior to the commencement of the study, formal permission was also obtained from the kindergarten principals and teachers. Parents were fully informed about the purpose and procedures of the research, and all provided written consent for their child’s participation. Participation was voluntary, and the participants received no incentives. The data collection process began with the administration of a sociometric test to all children in each classroom, enabling the classification of each child into a specific social profile category. Following this, 10 children from each class were randomly selected to form the final study sample. These children subsequently completed the Thinking Creatively in Action and Movement (TCAM) test, designed to assess motor creativity. Their responses were evaluated and scored based on Torrance’s standardized scoring tables, yielding individual scores for fluency, originality, and imagination. Lastly, the kindergarten teachers completed the Children’s Playfulness Scale for each participating child, based on their observed play behavior.

### 2.4. Data Analysis

Statistical analysis of the empirical data was conducted using SPSS version 29. Descriptive statistics were performed, including measures of central tendency and internal consistency (e.g., Cronbach’s alpha) for the subscales of the questionnaires. Correlation analyses were conducted to examine the relationships among the variables related to motor creativity, playfulness, and social profile. Subsequently, a two-step hierarchical regression analyses were performed to evaluate the study’s research hypotheses. In Step 1, the five dimensions of playful behavior, namely physical spontaneity, cognitive spontaneity, social spontaneity, sense of humor, and manifestation of joy, were entered as independent variables. In Step 2, the social profile variable was added to the model.

## 3. Results

The descriptive statistics of the variables, the skewness and kurtosis indices, and the Cronbach’s α internal consistency indices are presented in Table 1. Cronbach’s α internal consistency coefficients were within acceptable levels (*α* > 0.68) for all variables measured. The results of the correlation analysis indicated low correlation or no correlation among the motor creativity and playfulness factors. Social profile showed a negative low correlation with imagination (Table 2).

### Determinants of Motor Creativity

To assess multicollinearity, we examined the variance inflation factor (VIF) and tolerance values for all predictors included in the regression models. The VIF values ranged from 1.02 to 2.99, and the tolerance values ranged from 0.33 to 0.97, indicating acceptable levels of multicollinearity [31,32]. The results of the analysis indicated that, in step 1, the playfulness variables were significant predictors of fluency, *AdjR*^2^ = 0.15, *F*(5, 199) = 7.05, *p* < 0.001. Specifically, the physical spontaneity showed a significant effect (*β* = 0.23) as did the social spontaneity (*β* = 0.25). Interestingly, sense of humor negatively predicted fluency (*β* = –0.39). In Step 2, the inclusion of the social profile variable did not significantly enhance the model’s predictive power, *AdjR*^2^ = 0.16, *F*(6, 199) = 6.25, *p* < 0.01, with social profile showing a non-significant contribution (*β* = 0.09).

With respect to originality, the results of the analysis indicated that, in Step 1, playfulness significantly predicted originality, *AdjR*^2^ = 0.13, *F*(5, 199) = 5.93, *p* < 0.001. In particular, social spontaneity positively predicted originality (*β* = 0.22), whereas sense of humor was a significant negative predictor (*β* = –0.33). The addition of social profile in Step 2 did not significantly enhance the model’s predictive power, *AdjR*^2^ = 0.14, *F*(6, 199) = 5.22, *p* < 0.001, with social profile itself not reaching statistical significance (*β* = 0.08).

The data analysis showed similar results for imagination. More specifically, playfulness variables significantly and positively predicted imagination, *AdjR*^2^ = 0.07, *F*(5, 199) = 3.15, *p* < 0.001. In Step 1, both the physical spontaneity and social spontaneity showed a significant and positive effect (*β* = 0.24 and *β* = 0.16, respectively). The addition of the social profile in Step 2 enhanced the model’s predictive ability, *AdjR*^2^ = 0.09, *F*(6, 199) = 3.49, *p* < 0.05. Social profile itself was a significant negative predictor of imagination (*β* = –0.15), indicating an inverse relationship (Table 3).

## 4. Discussion

The present study was designed to investigate how preschoolers’ playfulness and social integration within peer groups influence their motor creativity. The results of the analyses indicated that playfulness dimensions have a statistically significant, but modest, association with motor creativity, but the social status of the child was not. More specifically, the results indicated that physical spontaneity and social spontaneity significantly predicted fluency in motor creativity, while sense of humor was a negative predictor. These findings align with the existing literature, suggesting that children who exhibit physical and social spontaneity are more likely to explore and generate diverse movement responses in open-ended tasks [18]. Physical spontaneity reflects an inclination toward active and uninhibited motor expression, which naturally supports the ability to produce a variety of motor solutions, which is core to fluency. Likewise, socially spontaneous children, who are comfortable engaging with peers, may benefit from collaborative play environments that encourage idea-sharing and imitation, fueling fluent motor behavior. These findings add nuance to our understanding of how intrinsic behavioral dispositions, namely physical and social spontaneity, influence creative movement, moving beyond general assumptions about playfulness. These insights help refine educational practices by highlighting which aspects of playfulness should be fostered to support diverse and fluent motor expression in early childhood.

Similar findings were observed with respect to originality. Social spontaneity emerged as a positive predictor of motor originality, while sense of humor again had a significant negative effect. These results reinforce the notion that social engagement during play fosters novel and unique movement responses, as children co-construct ideas and adapt creatively to others’ behaviors [10]. In socially rich play contexts, children are exposed to diverse motor models and are more likely to deviate from conventional patterns. This supports the literature that positions peer interaction and cooperative play as essential to the development of originality in motor behavior [11]. These findings advance the understanding of motor creativity by emphasizing the role of social spontaneity in fostering originality. They highlight how socially engaged children, through interactive play and peer collaboration, are more likely to generate novel movement responses. This underscores the value of cooperative, socially dynamic play environments in nurturing motor originality in early childhood.

However, the negative association between sense of humor and motor creativity challenges assumptions that playful teasing and joking necessarily promote divergent thinking. Humor and creative generation initially involve comparable neural activity patterns [33] and were expected to be associated. The negative association found in our study may suggest that, for young children, humor might be more about obtaining attention or being silly with peers rather than displaying flexible thinking. The consistent negative relationship between sense of humor and motor creativity dimensions may indicate that humorous behavior in young children, when not contextually aligned with physical tasks, could distract from goal-directed motor exploration. If this is the case, educators need to scaffold environments that balance physical engagement and structured social interaction while remaining sensitive to the type of playfulness being expressed.

Importantly, children’s social status did not predict fluency and originality, in contrast to evidence suggesting that peer interactions are associated with the development of motor creativity [10]. Also, this finding was inconsistent with the finding pertaining to the positive relation of social spontaneity with motor creativity dimensions. In this respect, it should be noted that social spontaneity reflects a child’s willingness to interact, share, and engage socially during play [34], regardless of how popular or accepted they are among peers. Children with high social spontaneity are more likely to be open, expressive, and responsive in group settings [35], which aligns closely with the demands of creative movement tasks. Such children may be comfortable initiating interactions, collaborating, and adapting ideas during play, which naturally leads to a greater number of responses (fluency) and more unique or varied actions (originality).

In contrast, social profile reflects a child’s status or popularity. That is, whether they are liked, disliked, or overlooked by peers [36]. While this can influence overall social experience, it does not necessarily capture how actively or confidently a child engages with others during play. A child might be well-liked (i.e., have a “popular” profile) but still be reserved or passive during physical play, contributing little to group motor tasks. On the flip side, a child who is not highly accepted might still be very socially spontaneous, actively participating and interacting with others. In addition, motor fluency and originality are largely individual behavioral outputs that benefit from interactive dynamics, but they do not depend on peer approval to occur. Social profile is an external peer-evaluated measure, while social spontaneity reflects an intrinsic behavioral disposition, which may explain why one relates more directly to motor creativity.

With respect to imagination, both physical spontaneity and social spontaneity emerged as positive predictors, with social profile emerging as a significant negative predictor. This outcome is consistent with previous research indicating that physically and socially engaged children are more likely to explore imaginative representations through movement [18,19]. Children who are physically spontaneous show comfort with bodily expression, essential for imaginative tasks like mimicking animals or portraying abstract concepts. Similarly, social spontaneity supports imagination by allowing children to borrow and build upon each other’s ideas in playful exchanges.

Notably, social status (as reflected in the sociometric profile) was negatively related to imagination. This finding contrasts with assumptions that socially accepted children have stronger self- and emotional regulation and are more expressive [37]. One possible explanation is that children with high social standing may feel a stronger need to conform to peer expectations or avoid behaviors that could be perceived as unusual, thereby limiting their willingness to take imaginative risks. In contrast, children with lower social status may experience fewer social pressures and may use imaginative play as a form of individual expression or coping strategy, engaging more freely in fantasy-based movement tasks. This interpretation is supported by the idea that creativity, particularly imagination, can flourish in children who are less focused on peer approval and more engaged in internal or solitary play modes [38]. Furthermore, the development of imagination is closely linked to social cognition and role-taking abilities. Children who are less integrated into peer groups may engage more in solitary imaginative activities, which can enhance their ability to understand different perspectives and foster creative thinking. This solitary play may offer a safe space for these children to explore ideas without the fear of social judgment, thereby nurturing their imaginative capacities [38].

The study offers a nuanced contribution to the field by distinguishing between children’s social status and their social spontaneity in relation to motor creativity. While social spontaneity positively predicted creative outcomes, social status did not predict fluency or originality and was even negatively associated with imagination. These findings challenge the common assumption that higher peer acceptance inherently supports creativity. Instead, they suggest that active, engaged social behavior, regardless of popularity, is more influential in fostering creative movement. This distinction refines our understanding of social dynamics in early childhood and emphasizes the importance of promoting inclusive, interactive play environments over peer-based hierarchies.

The present findings hold significant implications for early childhood education and the promotion of holistic child development. First, the demonstrated predictive value of physical and social spontaneity on motor creativity highlights the importance of fostering these traits through well-designed learning environments. Educators should intentionally integrate activities that allow children to express themselves freely through movement, such as open-ended play scenarios, guided dance, creative locomotion, and role-play games. These activities not only stimulate diverse motor outputs (fluency and originality) but also provide opportunities for children to collaborate, imitate, and innovate, which are key processes in creative development. Furthermore, the differential role of social spontaneity and social status offers crucial insight into peer interactions in early childhood settings. Educators should focus less on promoting popularity or structured social rankings and more on nurturing socially inclusive climates that value initiative, peer responsiveness, and expressive freedom. Purposeful group activities that rotate partners and roles, celebrate individual expression, and provide emotionally safe spaces can reduce conformity pressures, particularly among highly accepted children, and boost creative engagement across all sociometric groups. In addition, the consistent negative relationship between sense of humor and motor creativity found in this study suggests that not all expressions of playfulness contribute equally to creative development. Educators should differentiate between types of playful behaviors, recognizing when humor becomes a distraction rather than a tool for engagement. Structured movement tasks that balance spontaneity with focus may help channel children’s energy toward goal-directed creative expression.

The findings of the present study open several valuable avenues for future research and practice in the field of early childhood education. First, future studies should adopt longitudinal designs to explore how playful dispositions and peer-related social dynamics influence motor creativity over time. A developmental perspective could clarify whether the predictive effects observed here remain stable, intensify, or change as children mature and gain more social and motor experience. Longitudinal data would also allow for stronger inferences regarding causality and the developmental trajectories of motor creativity. Second, future research would benefit from incorporating multi-informant approaches, including parental reports, child self-reports, and direct observational tools, to triangulate behavioral data and enhance measurement validity. Similarly, observational assessments of children’s behavior during actual play contexts could offer richer insights into how spontaneity and social engagement unfold in real time. Third, future research could explore whether children with higher peer status experience stronger conformity pressures or reduced willingness to engage in solitary imaginative activities. Investigating the role of internalized peer expectations, classroom climate, and teacher attitudes could shed light on how social contexts either constrain or support imaginative expression. Fourth, the modest zero-order correlations indicate small effect sizes, which suggest that, while certain dimensions of playfulness contribute to motor creativity, other unmeasured factors are likely involved. This also reinforces the need for future studies to explore broader or more integrated models, including contextual, cognitive, or emotional predictors, to better explain the variance in motor creativity. Finally, future studies could examine how digital or augmented play environments might influence the development of motor creativity and social engagement in preschoolers. As technology becomes increasingly integrated into early education, it is important to understand whether these tools support or hinder the development of spontaneous, embodied, and socially interactive play behaviors.

## 5. Conclusions

Despite its valuable contributions, this study has several limitations. First, the use of teacher-reported measures for assessing playfulness may introduce subjective bias, potentially influencing the reliability of the results. Second, the cross-sectional design limits the ability to infer causal relationships between playfulness, social status, and motor creativity. Additionally, the sample was limited to one geographic region in Greece, which may affect the generalizability of the findings to broader populations. Future studies should consider longitudinal designs and incorporate observational or multi-informant data to strengthen validity. In conclusion, this study highlights the pivotal role of playfulness, particularly physical and social spontaneity, in enhancing preschoolers’ motor creativity. While social profile did not significantly predict fluency or originality, its negative association with imagination suggests complex social–emotional dynamics that are worth further exploration. These findings emphasize the need for inclusive, playful educational environments that foster spontaneous movement, creativity, and social engagement, supporting holistic child development.

## Figures and Tables

**Table 1:** Descriptive statistics and internal consistency indices for the subscales of the questionnaires.

	Mean	Standard Deviation	Skewness	Kurtosis	Cronbach *α*
Fluency	113.84	35.83	0.62	−0.87	-
Originality	112.33	27.88	0.77	−0.61	-
Imagination	96.33	10.77	−0.42	0.09	-
Physical spontaneity	3.87	0.69	−0.79	0.39	0.80
Cognitive spontaneity	2.98	0.76	−0.21	−0.25	0.75
Social spontaneity	3.54	0.67	−0.41	0.45	0.78
Sense of humor	2.80	0.70	−0.26	−0.00	0.68
Manifest joy	3.89	0.61	−0.49	0.17	0.76
Social profile	2.74	1.26	0.20	−1.00	-

**Table 2:** Pearson correlation coefficient for all subscales of the questionnaires.

	2	3	4	5	6	7	8	9
1. Fluency	0.96 **	0.26 **	0.16 *	0.05	0.24 **	−0.09	0.14 *	0.08
2. Originality		0.23 **	0.17 *	0.08	0.25 *	−0.04	0.16 *	0.07
3. Imagination			0.21 **	0.14 *	0.18 **	0.18 **	0.14 *	−0.17 *
4. Physical spontaneity				0.70 *	0.31 **	0.62 **	0.74 **	0.02
5. Cognitive spontaneity					0.33 **	0.67 **	0.57 **	−0.00
6. Social spontaneity						0.29 **	0.41 **	−0.12
7. Sense of humor							0.64 **	−0.02
8. Manifest joy								0.04
9. Social profile								

Note: ** *p* < 0.001, * *p* < 0.05.

**Table 3:** Prediction of motor creativity from playful behavior and social profile.

		Fluency			Originality			Imagination		
		b	t	sig	b	t	sig	b	t	sig
Step 1	Physical spontaneity	0.25	2.23	0.02	0.21	1.82	0.07	0.24	2.02	0.04
	Cognitive spontaneity	0.00	0.02	0.98	0.01	0.16	0.86	−0.09	−0.84	0.40
	Social spontaneity	0.23	3.14	0.00	0.22	3.05	0.00	0.16	2.10	0.03
	Sense of humor	−0.39	−4.02	0.00	−0.33	−3.33	0.00	0.15	1.44	0.14
	Manifest joy	0.11	1.04	0.29	0.12	1.11	0.26	−0.15	−1.31	0.18
Step 2	Physical spontaneity	0.25	2.22	0.02	0.21	1.81	0.07	0.24	2.06	0.04
	Cognitive spontaneity	0.00	0.01	0.99	0.01	0.15	0.87	−0.09	−0.83	0.40
	Social spontaneity	0.24	3.33	0.00	0.24	3.21	0.00	0.13	1.77	0.07
	Sense of humor	−0.39	−3.94	0.00	−0.32	−3.25	0.00	0.13	1.33	0.18
	Manifest joy	0.10	0.91	0.36	0.11	1.00	0.31	−0.12	−1.12	0.26
	Social profile	0.09	1.43	0.15	0.08	1.26	0.20	−0.15	−2.20	−0.02

## Data Availability

The data are available from the corresponding author upon request.

## References

[1] Ginsburg K.R., American Academy of Pediatrics Committee on Communications, American Academy of Pediatrics Committee on Psychosocial Aspects of Child and Family Health (2007). The Importance of Play in Promoting Healthy Child Development and Maintaining Strong Parent-Child Bonds. Pediatrics.

[2] Ledford J.R., Pustejovsky J.E. (2023). Systematic Review and Meta-Analysis of Stay-Play-Talk Interventions for Improving Social Behaviors of Young Children. J. Posit. Behav. Interv..

[3] Karaca N.H., Uzun H., Metin Ş. (2020). The Relationship between the Motor Creativity and Peer Play Behaviors of Preschool Children and the Factors Affecting This Relationship. Think. Skills Creat..

[4] Houser N., Woolley A., Kriellaars D. (2024). An Exploration of Physical Literacy and Movement Creativity. Circus Arts Life Sci..

[5] Rudd J.R., Pesce C., Strafford B.W., Davids K. (2020). Physical Literacy—A Journey of Individual Enrichment: An Ecological Dynamics Rationale for Enhancing Performance and Physical Activity in All. Front. Psychol..

[6] D’Souza A.A., Wiseheart M. (2018). Cognitive Effects of Music and Dance Training in Children. Arch. Sci. Psychol..

[7] Neville R.D., Makopoulou K. (2020). Effect of a Six-Week Dance-Based Physical Education Intervention on Primary School Children’s Creativity: A Pilot Study. Eur. Phys. Educ. Rev..

[8] Santos S., Jiménez S., Sampaio J., Leite N. (2017). Effects of the Skills4Genius Sports-Based Training Program in Creative Behavior. PLoS ONE.

[9] Ourda D., Gregoriadis A., Mouratidou K., Grouios G., Tsorbatzoudis H. (2017). A Motor Creativity Intervention in the Greek Early Childhood Education Settings: Effects on Beliefs about Health. J. Early Child. Educ. Res..

[10] Bicenturk F. (2024). Investigating Benefits of Creative Dance on Cognitive, Social and Creative Development with Children Aged 6–7 Years. Ph.D. Dissertation.

[11] Garaigordobil M., Berrueco L., Celume M.P. (2022). Developing Children’s Creativity and Social-Emotional Competencies through Play: Summary of Twenty Years of Findings of the Evidence-Based Interventions “Game Program”. J. Intell..

[12] Pesce C., Masci I., Marchetti R., Vazou S., Saakslahti A., Tomporowski P.D. (2016). Deliberate Play and Preparation Jointly Benefit Motor and Cognitive Development: Mediated and Moderated Effects. Front. Psychol..

[13] Proyer R.T., Tandler N., Brauer K., Guendouzi S., Ellis A.P.J., Grainger A. (2019). Playfulness and Creativity: A Selective Review. Creativity and Humor.

[14] Barnett L.A. (1991). The Playful Child: Measurement of a Disposition to Play. Play Cult..

[15] Aartun I., Lambert K., Walseth K. (2023). How Pupils’ Playfulness Creates Possibilities for Pleasure and Learning in Physical Education. Phys. Educ. Sport Pedagog..

[16] Boon B., Rozendaal M.C., Van den Heuvel-Eibrink M.M., van der Net J., van Grotel M., Stappers P.J. (2020). Design Strategies for Promoting Young Children’s Physical Activity: A Playscapes Perspective. Int. J. Des..

[17] Adamopoulou E., Karatrantou K., Kaloudis I., Krommidas C., Gerodimos V. (2024). An Effective and Playful Way of Practicing Online Motor Proficiency in Preschool Children. Children.

[18] Trevlas E., Matsouka O., Zachopoulou E. (2003). Relationship between Playfulness and Motor Creativity in Preschool Children. Early Child Dev. Care.

[19] Lee-Cultura S., Sharma K., Giannakos M. (2022). Children’s Play and Problem-Solving in Motion-Based Learning Technologies Using a Multi-Modal Mixed Methods Approach. Int. J. Child-Comput. Interact..

[20] Ma X., Yang N., Huang M., Zhan S., Cao H., Jiang S. (2024). Relationships between Gross Motor Skills, Psychological Resilience, Executive Function, and Emotional Regulation among Chinese Rural Preschoolers: A Moderated Mediation Model. Heliyon.

[21] Fernández J.E.R., Pereira V., Pereira B. (2024). The Playground as a Laboratory for Playful Practices and Social Relations. Retos Nuevas Tend. Educ. Fis. Deporte Recreacion.

[22] Cavanaugh D.M., Clemence K.J., Teale M.M., Rule A.C., Montgomery S.E. (2017). Kindergarten Scores, Storytelling, Executive Function, and Motivation Improved through Literacy-Rich Guided Play. Early Child. Educ. J..

[23] D’Adamo P., Lozada M. (2024). Playful Enactive Interventions Can Enhance Agency, Empathy and Social Integration in Children. Learn. Instr..

[24] Yildirim Y., Yilmaz Y. (2023). Promoting Creativity in Early Childhood Education. PLoS ONE.

[25] Salaj S., Masnjak M. (2022). Correlation of Motor Competence and Social-Emotional Wellbeing in Preschool Children. Front. Psychol..

[26] Fantuzzo J., Sekino Y., Cohen H.L. (2004). An Examination of the Contributions of Interactive Peer Play to Salient Classroom Competencies for Urban Head Start Children. Psychol. Sch..

[27] Boni C., Zancan S., Diehl R., Flores J.L.d.S., Faccin T.R., Moreschi M.P., Lima M.S.d.S. (2023). The Importance of Playing in Childhood Education. Rev. Genero Interdiscip..

[28] Barnett L.A. (1990). Playfulness: Definition, design, and measurement. Play. Cult..

[29] Zachopoulou E., Makri A., Pollatou E. (2009). Evaluation of Children’s Creativity: Psychometric Properties of Torrance’s ‘Thinking Creatively in Action and Movement’ Test. Early Child Dev. Care.

[30] Moreno J. (1934). Who Shall Survive? A New Approach to the Problem of Human Interrelations.

[31] Hair J.F., Black W.C., Babin B.J., Anderson R.E. (2010). Multivariate Data Analysis.

[32] Kutner M.H., Nachtsheim C.J., Neter J., Li W. (2005). Applied Linear Statistical Models.

[33] Sun C., Zhou Z. (2024). Electroencephalography (EEG) Evidence for the Psychological Processes of Humor Generation: A Comparison Perspective on Humor and Creativity. Behav. Sci..

[34] Fung W.K., Chung K.K.H. (2023). Playfulness as the Antecedent of Kindergarten Children’s Prosocial Skills and School Readiness. Eur. Early Child. Educ. Res. J..

[35] Coplan R.J., Armer M. (2007). A “Multitude” of Solitude: A Closer Look at Social Withdrawal and Nonsocial Play in Early Childhood. Child Dev. Perspect..

[36] Cañas E., Estevez E., Estevez J.F. (2022). Sociometric Status in Bullying Perpetrators: A Systematic Review. Front. Commun..

[37] Hay D.F., Payne A., Chadwick A. (2004). Peer Relations in Childhood. J. Child Psychol. Psychiatry.

[38] Gleason T.R. (2004). Imaginary Companions and Peer Acceptance. Int. J. Behav. Dev..

